# The effects of increasing and decreasing dopamine on inhibition of return: a psychopharmacological investigation

**DOI:** 10.1007/s00221-026-07355-1

**Published:** 2026-09-27

**Authors:** Grace A. Whitaker, Ellen Poliakoff, Sarah L. Martin, Joanna C. Neill, Monty Silverdale, Christopher Kobylecki, Wael El-Deredy

**Affiliations:** 1 Department of Electronic Engineering and Advanced Center for Electrical and Electronic Engineering, Universidad Técnica Federico Santa Maria, Valparaiso, Chile; 2 Chair of Acoustics and Haptics, Technical University of Dresden, Dresden, Germany; 3 Andrew Mayes Centre for Cognitive Neuroscience, Division of Psychology, Communication and Human Neuroscience, The University of Manchester, Manchester, UK; 4 Translational and Computational Neurosciences Unit, Department of Psychology, Faculty of Health & Education, Manchester Metropolitan University, Manchester, UK; 5 Division of Pharmacy & Optometry, Faculty of Biology, Medicine and Health, Manchester Academic Health Science Centre, School of Health Sciences, University of Manchester, Manchester, UK; 6 Department of Neurology, Manchester Academic Health Science Centre, Salford Royal Foundation Trust, University of Manchester, Manchester, UK; 7 Department of Neurology, Manchester Centre for Clinical Neurosciences, Northern Care Alliance NHS Foundation Trust, Salford, UK; 8 Division of Neuroscience, Faculty of Biology, Medicine and Health, School of Biological Sciences, The University of Manchester, Manchester, UK; 9 Brain Dynamics Lab, Interdisciplinary Center of Biomedical and Engineering Research for Health, Universidad de Valparaíso, Valparaíso, Chile

**Keywords:** Inhibition of return, Dopamine, Striatum, D2-receptors, Attentional mechanisms

## Abstract

Inhibition of Return (IOR) is the phenomenon of being slower to re-attend to previously attended locations, and is thought to improve the efficiency of attention. Previous studies of dopaminergic disorders indicate that excessive or insufficient dopamine signaling in the striatum of the brain reduces IOR magnitude. However, there have been no studies of the direct effects of dopamine depletion on IOR in healthy individuals, or that have compared the effect of dopamine increases and decreases within the same population. Therefore, in the present study, we administered a selective dopamine-2 receptor (D2R) agonist and antagonist to the same healthy participants (cabergoline and amisulpride, respectively) and measured IOR. We further investigated the effects of dopamine manipulation on the underlying cognitive processes of IOR using event-related potentials. Both manipulations reduced the IOR effect relative to placebo, consistent with a non-linear relationship between dopamine and IOR. Given the D2R selectivity of both drugs and the accompanying changes in eye-blink rate, these effects are likely driven by primarily striatal dopamine, potentially reflecting frontostriatal modulation of the balance between attentional flexibility and stability.

**Supplementary Information:**

The online version contains supplementary material available at https://doi.org/10.1007/s00221-026-07355-1.

## Introduction

The ability to attend to relevant stimuli in the environment is crucial, as our brains receive more information than they can process (Oberauer 2019; Bari and Robbins 2013). The importance of this ability is highlighted by disorders in which attentional filtering is disrupted, resulting in unwanted or inefficient cognitive processing, for example in patients with Schizophrenia (Luck et al. 2019; Hahn et al. 2022), Parkinson’s disease (Aarsland et al. 2021) and high trait impulsivity (Nigg 2013). It is thought that the neurotransmitter dopamine is key for attentional inhibitory processes (Bari and Robbins 2013; Westbrook et al. 2020). Hence in the present study, we investigate the role of dopamine in Inhibition of Return (IOR)—a mechanism for the attentional inhibition of irrelevant stimuli (Klein 2000; Lupiáñez et al. 2006).

IOR was first observed as the delayed response to visual targets appearing in the same location as a previous cue (“cued location”), compared to a novel uncued location (Posner and Cohen 1984). The effect is apparent for specific objects, even when moved in space (Tipper et al. 1991), acts over a spatial gradient strongest at the cued location itself (Samuel and Kat 2003), and is significantly weaker when the initial context is altered (Klein and Macinnes 1999). Theoretical work has further refined our understanding of IOR as an information processing phenomenon with distinct computational properties (Tsotsos 2021; Redden et al. 2021), with empirical evidence continuing to support its role as a foraging facilitator in visual search contexts (Li et al. 2023; Klein et al. 2023).

Since its conception, researchers have studied the neural substrates of IOR, establishing a central role of the midbrain superior colliculus (SC) (Dorris et al. 2002; Rafal et al. 1988; Sapir et al. 1999). In general, the SC is considered a crucial structure for visual orienting behaviour, containing neurons which encode visual saliency maps (White et al. 2017). For IOR specifically, the SC is thought to generate inhibitory ‘tags’, which are then transmitted to frontal cortical brain regions to be maintained (Fecteau and Munoz 2005; Klein 2000).

Further studies into the structure and function of the SC provide additional context for its role in IOR. As an evolutionarily ancient structure, the SC has sophisticated mechanisms for integrating sensory information and motor output (Guillamón-Vivancos et al. 2024; Hoy and Farrow 2025), with deeper layers being particularly important for attentional modulation that may underlie inhibitory processes like IOR (Ding et al. 2019). These connections have been functionally linked to the processing of attentional salience and subsequent reinforcement learning (Redgrave et al. 2010).

Comparative studies have shown that while both the frontal eye field and SC contribute to covert spatial attention, they do so through distinct computational mechanisms (Bollimunta et al. 2018), suggesting that the SC's role in IOR may involve specialised circuits that differ from those supporting other forms of attention. Crucially, the SC is believed to exert some of its orienting effects via midbrain dopamine signalling, as the SC provides afferent signals to the dopaminergic neurons in the substantia nigra and dopamine cell groups in the ventral midbrain (Dommett et al. 2005; Ikemoto et al. 2015; Redgrave et al. 2010).

Evidence linking dopamine to IOR comes from multiple sources. Table 1 summarises key findings across clinical populations, genetic studies, and pharmacological manipulations. Populations with reduced dopamine (Parkinson's disease, cocaine users with low dopamine-2 receptor (D2R) availability) as well as those with presumed elevated dopamine (schizophrenia) demonstrate diminished IOR relative to healthy controls. However, the pattern for acute pharmacological manipulations is more complex. Whilst *d*-amphetamine enhanced IOR (Fillmore et al. 2005), the D2R agonist bromocriptine produced opposite effects depending on dopamine transporter DAT1 genotype – with individuals presumed to have lower baseline dopamine showing increased IOR and those with higher baseline showing decreased IOR (Rokem et al. 2012). This interaction between genotype and drug suggests a non-linear relationship between striatal dopamine and IOR.

**Table 1:** Summary of previous research relating dopamine to Inhibition of Return (IOR)

Study	Population/Manipulation	DA State	IOR Type	Finding	Effect
Poliakoff et al. (2003)	Parkinson's disease	Low	Tactile	Reduced	↓
Possin et al. (2009)	Parkinson's disease	Low	Visual Location	Reduced	↓
			Visual Object	Intact	—
Gouzoulis-Mayfrank et al. (2004)	Schizophrenia	High	Visual	Reduced	↓
Gouzoulis-Mayfrank et al. (2007)	Schizophrenia	High	Visual	Reduced	↓
	Prodromal subjects	High	Visual	Intact	—
Okada et al. (2021)	Schizophrenia	High	Free-viewing	Reduced	↓
Wu et al. (2025)	Schizophrenia	High	Location-based	Reduced	↓
			Object-based	Intact	—
Colzato and Hommel (2009)	Cocaine users (low D2Rs)	Low	Visual	Reduced	↓
Bucker and Theeuwes (2014)	High trait impulsivity (low D2Rs)	Low	Visual	Reduced	↓
Fillmore et al. (2005)	Healthy adults + *d*-amphetamine	Increased	Visual	Enhanced	↑
Colzato et al. (2010)	DAT1 9-repeat carriers	Higher	Visual	Enhanced	↑
	DAT1 10-repeat carriers	Lower	Visual	Reduced	↓
Rokem et al. (2012)	Bromocriptine + DAT1 9-repeat carriers	Increased from lower baseline	Visual	Enhanced	↑
	Bromocriptine + DAT1 10-repeat carriers	Increased from higher baseline	Visual	Reduced	↓

↓ = reduced IOR; ↑ = enhanced IOR; — = no effect or intact IOR

Beyond IOR specifically, converging evidence supports that striatal dopamine plays a broader role in attentional processes. Longitudinal studies in Parkinson's disease show that striatal dopamine levels correlate with performance on attentional and executive function tasks such as trail-making and fluency tests (Fornari et al. 2021). Visual selective attention deficits, measured by the Visual Search Test, are present even in early-stage Parkinson's disease prior to dopaminergic medication (Cammisuli et al. 2021; Chung et al. 2018), with performance correlating directly with dopamine transporter availability in the caudate nucleus (Cammisuli et al. 2021). These findings suggest that dopamine depletion, rather than medication effects or broader neuropathological changes, underlies attentional impairments in people with Parkinson's disease.

D2Rs appear particularly relevant for attentional control. Allelic variation in the D2R gene is associated with attentional impulsiveness (Taylor et al. 2018), while polygenic scores indexing DRD2-related co-expression networks correlate with striatal dopamine function (D'Ambrosio et al. 2022). In clinical populations, D2R genetic variants are associated with attention-deficit hyperactivity disorder (ADHD) in children (Moro et al. 2019), highlighting the broader relevance of D2R variations for attentional control across development.

Further research supports the distinct roles of dopamine receptor-1 (D1R) versus D2Rs in attentional control. Whilst D1R signaling specifically protects mental representations from distracting bottom-up influences, D2Rs appear more involved in cognitive flexibility and decision-making processes (Bensmann et al. 2020; Verharen et al. 2019). Importantly, pharmacological studies show that D1R and D2R blockades in the prefrontal cortex produce attentional impairments through different neuronal circuits (Wulaer et al. 2021), supporting the specific relevance of D2Rs for the type of attentional flexibility that may underlie IOR.

This type of inverted-U function has been observed for dopamine levels in other domains, such as working memory and cognitive control (Cools and D'Esposito 2011; Mattay et al. 2000). However, as noted by Floresco et al. (2013), the optimal balance between D1 and D2R stimulation can produce various non-linear relationships depending on the task, including quadratic, cubic, or other functional forms. Spencer et al. (2024) directly demonstrated that cognition-enhancing versus cognition-impairing doses of psychostimulants exert opposing actions on frontostriatal neural coding during working memory tasks, providing neurophysiological support for inverted-U relationships in dopaminergic cognitive control. The complexity of dopamine's role is further illustrated by findings that dopamine promotes cognitive effort by making the benefits of tasks more rewarding compared to the cost of cognitive work (Westbrook et al. 2020), and that dopaminergic effects on cognitive control processes can vary by individual genotype (Furman et al. 2020). Specifically, dopamine appears to guide competition for cognitive control through common mechanisms affecting both working memory and response conflict (Fallon et al. 2019), suggesting that the non-linear relationship observed in these domains may extend to attentional inhibition mechanisms like IOR.

However, evidence for non-linear effects of dopamine on IOR remains incomplete. Firstly, decreases in dopamine signalling have yet to be directly studied – having only been inferred from people with Parkinson’s disease (Poliakoff et al. 2003; Possin et al. 2009). In Parkinson's disease, several brain structures show damage before the dopaminergic neurons of the substantia nigra in the basal ganglia (BG) are affected (Horsager and Borghammer 2024; Ayano 2016). Therefore, any effects of Parkinson’s disease on IOR cannot be solely attributed to reductions in striatal dopamine. Furthermore, IOR was studied in people with Parkinson’s disease taking various forms of dopaminergic medication in which is likely to have affected attentional control (cf, Riedel et al. 2022) and thus the measurement of IOR.

Secondly, the dopamine agonists used in previous studies (*d*-amphetamine and bromocriptine) have several non-selective effects on dopaminergic, adrenergic, and serotonergic systems (Heal et al. 2013; Millan et al. 2002). Consequently, their effects on IOR may, at least in part, be the result of other neurotransmitters or receptors.

Therefore, in the present study, we investigate the direct, causal effects of both increased and decreased striatal dopamine signalling on IOR. To target the striatum, we utilised drugs with high affinity and selectivity for D2Rs. Cabergoline was selected as the D2 agonist due to its considerably higher affinity for D2 receptors compared to other commonly used dopamine agonists, such as bromocriptine and ropinirole, combined with good tolerability (Gerlach et al. 2003). The dose of 1.25mg was chosen based on previous studies demonstrating significant cognitive effects in healthy participants (Frank and O'Reilly 2006; Nandam et al. 2013) and exceeds the typical starting dose for patients with Parkinson's disease (Del Dotto and Bonuccelli 2003). Amisulpride was selected as the D2 antagonist due to its higher affinity for D2Rs compared to haloperidol and comparable affinity to sulpiride, while producing fewer side effects (Tardieu et al. 2003; Delcker et al. 1990). The dose of 400mg has been used successfully in previous cognitive studies with healthy volunteers (Chung et al. 2012; Perrault et al. 1997). Both doses were selected to ensure postsynaptic rather than presynaptic receptor stimulation (Frank and O’Reilly 2006). Both drugs have sufficiently long elimination half-lives to maintain stable plasma levels during behavioral testing (see Methods “Pre-task” section for pharmacokinetic details).

Another issue of the aforementioned pharmacological studies is the lack of verification checks that manipulations did indeed increase or decrease dopamine signalling. This is important as at low dosages, dopaminergic drugs bind to presynaptic rather than postsynaptic dopamine receptors. Stimulation of presynaptic receptors has an opposing effect on dopamine signalling compared to postsynaptic receptors (Frank and O’Reilly 2006). In other words, it is difficult in such studies to ascertain if there was an increase or decrease in dopamine signalling (as acknowledged by Rokem et al. 2012). In order to avoid presynaptic effects in the present study, 1.25mg of cabergoline and 400mg of amisulpride were utilised. These dosages have been found to be clinically effective in increasing and decreasing dopamine transmission to treat the symptoms of Parkinson’s disease and Schizophrenia, respectively (Del Dotto and Bonuccelli 2003; McKeage and Plosker 2004), thus suggesting robust postsynaptic effects.

Spontaneous eye-blink rate (EBR) can be used as an indirect measure of striatal dopamine, as it has been found that individuals blink more when striatal dopamine levels are higher (Jongkees and Colzato 2016). This has been shown for baseline dopamine levels (Agostino et al. 2008; Kowal et al. 2011; Zhang et al. 2015), and the effects of pharmacological manipulations (Blin et al. 1990; Depue et al. 1994; Kleven and Koek 1996). This effect is believed to be mediated by the influence of the BG and SC on the trigeminal spinal nucleus (via the trigeminal nerve)—which usually relays information regarding the present state of the cornea to regulate blinking (Kaminer et al. 2011; Maffei and Angrilli 2018). Therefore, in the present study, EBR was utilised as a non-invasive measure of striatal dopamine manipulations.

It should be noted that the relationship between EBR and dopamine signaling remains debated, with recent neuroimaging studies failing to find correlations between EBR and dopamine measures in healthy adults (Dang et al. 2017; Sescousse et al. 2018; van den Bosch et al. 2023), though pharmacological studies continue to show EBR sensitivity to dopamine manipulations (reviewed in Jongkees and Colzato 2016). Nevertheless, EBR represents the only practical, non-invasive metric available for monitoring central dopaminergic effects during behavioral testing. We therefore utilised EBR as a manipulation check while acknowledging its limitations (see Discussion “Eye-blink rate as an index of dopaminergic effects” section).

While cabergoline and amisulpride have high affinity for D2Rs and are expected to affect striatal dopamine signaling, D2Rs are also widely expressed in cortical regions, particularly prefrontal cortex. Cortical D2Rs play crucial roles in cognitive stability and flexibility (Durstewitz and Seamans 2008), and evidence demonstrates that D1 and D2 blockade in prefrontal cortex produce distinct attentional impairments through different neuronal circuits (Wulaer et al. 2021). Therefore, while we expect our pharmacological manipulations to affect striatal dopamine signaling, cortical D2 modulation likely also contributes to any observed effects on attention.

To gain a deeper understanding of the cognitive processes underlying IOR and investigate whether dopaminergic manipulations affect early sensory versus later selective attentional stages, we employed event-related potentials (ERPs) obtained from electroencephalography (EEG). While EEG cannot directly measure subcortical striatal activity, it provides a window into cortical processing consequences of dopaminergic manipulation. Given that D2Rs modulate cortical attentional processes (Durstewitz and Seamans 2008; Wulaer et al. 2021), and that IOR involves both subcortical (SC) and cortical (prefrontal) mechanisms, ERPs are well-suited to reveal how dopamine affects the cortical stages of attentional selection in IOR.

A notable debate in the literature concerns whether IOR results from alterations in early sensory orienting or later selective attentional processes. Previous research has identified specific ERP components associated with IOR: the P1, which is smaller for cued compared to uncued targets, indicating that IOR emerges at the sensory stage of orienting (McDonald et al. 1999; Satel et al. 2013), and the N2pc, which shows smaller amplitudes for cued compared to uncued targets, indicating the involvement of selective attention (McDonald et al. 2008). This temporal distinction may map onto the anatomical dissociation between subcortical and cortical IOR mechanisms. Early sensory modulation may reflect SC processing (Sapir et al. 1999; Dorris et al. 2002), while later selective attention effects may reflect cortical control processes, potentially including the PFC (Prime and Ward 2006; Badre 2025). Given that dopamine modulates both subcortical and cortical pathways via D2Rs (Ott and Nieder 2019; Gerlach et al. 2003), we reasoned that measuring both early (P1) and late (N2pc) ERP components may provide some insight into which processing stages, and by inference, which anatomical pathways may be modulated by D2R manipulation.

Advanced ERP analyses have demonstrated that IOR involves a complex temporal sequence where initial selection processes may be delayed but subsequently enhanced, particularly affecting the N2pc component (Lin et al. 2020). Further research has revealed more nuanced effects, with increased IOR task difficulty modulating both P1 and N2pc components (Li et al. 2018). These findings suggest that dopaminergic manipulations may differentially affect early versus late stages of the attention selection process. Nevertheless, it remains to be seen how striatal dopamine may influence stages of attentional orienting, as previous studies were purely behavioural in nature. As such, in the present study, EEG was utilised to measure how sensory and selective attentional ERPs in IOR (P1 and N2pc, respectively) are influenced by alterations in dopamine signalling.

In summary, there is evidence that dopamine is important for IOR. However, questions remain regarding (1) the direct effect of increased and decreased dopamine within the same individuals, (2) the specific involvement of D2Rs, and (3) the processing stages involved. The present study aims to investigate these issues by measuring IOR with (1) a dopamine antagonist (as well as agonist), (2) drugs with a high affinity for D2Rs, and (3) EEG brain imaging combined with a target-target paradigm. The latter involves responding to every stimulus such that there is no requirement to inhibit motor responses, thus avoiding motoric confounds in true attentional IOR (e.g. Welsh and Pratt 2006; Poliakoff et al. 2007). Lastly, spontaneous EBR will be employed as an outcome measure for the pharmacological manipulations employed.

We hypothesised that the size of the IOR effect may show a non-linear relationship (greatest IOR in the placebo condition) or increase linearly with striatal dopamine level (greatest IOR in the agonist condition, smallest IOR in the antagonist condition). While the existing literature could support either hypothesis, the linear hypothesis might seem more parsimonious given evidence that dopamine increases generally enhance attentional control. However, the interaction between genotype and drug observed by Rokem et al. (2012), where bromocriptine effects on IOR depended on baseline dopamine levels, suggested the possibility of a non-linear relationship. Alterations in behavioural IOR may be reflected by alterations in sensory orienting (P1) and/or selective attention (N2pc) processing stages.

## Method

### Design

A double-blind, placebo-controlled, repeated measures, triple crossover design was employed. Across three visits separated by at least one week, participants were orally administered either cabergoline (1.25 mg), amisulpride (400 mg), or placebo (sugar pill). Each visit consisted of identical procedures outlined below.

The study was approved by the University of Manchester Research Ethics Committee and was further approved by the UK Health Research Authority for the use of a National Health Service site (Salford Royal Hospital).

### Recruitment and screening

Individuals were recruited via university email announcements, public study participation websites, and printed posters around the University of Manchester, UK. In order to avoid recruiting individuals extremely high or low in trait impulsivity, prospective participants completed an online version of the Barratt Impulsiveness Scale (BIS-11, Patton and Stanford 1995). Participants were then further health-screened in person by a clinician.

### Participants

Thirty-one participants were deemed eligible to participate in the full study (2 left-handed; 16 female; mean age 22.4 years; standard deviation 3.2 years). All participants gave written informed consent and received financial compensation for their participation.

### Procedures

#### Pre-task

Both drugs utilised in this study reach peak plasma levels approximately two hours following oral administration, though with slightly different time courses: cabergoline reaches maximum plasma concentration (Tmax) at 2–3 h with an extended elimination half-life (t½ = 63–109 h; Del Dotto and Bonuccelli 2003), while amisulpride reaches Tmax at 1–4 h with a plasma elimination half-life (t½) of 12 h (Mauri et al. 2014; Rosenzweig et al. 2002). The 2-h delay ensures that both drugs were at or near peak plasma concentration during testing, and the half-lives of both compounds ensured stable plasma levels throughout the approximately 30-min testing session. The identical 2-h delay across all three conditions maintained the integrity of the double-blind design. Therefore, following drug administration, participants remained in a neutral environment for two hours before testing procedures began. During this time, EEG scalp electrodes were connected.

Spontaneous eye-blinks were then measured for nine minutes. During this time, participants were asked to rest (without closing their eyes) whilst the EEG electrodes recorded their ‘resting activity’. Participants then completed the computerised IOR task described below.

#### IOR task

**Fig. 1:**
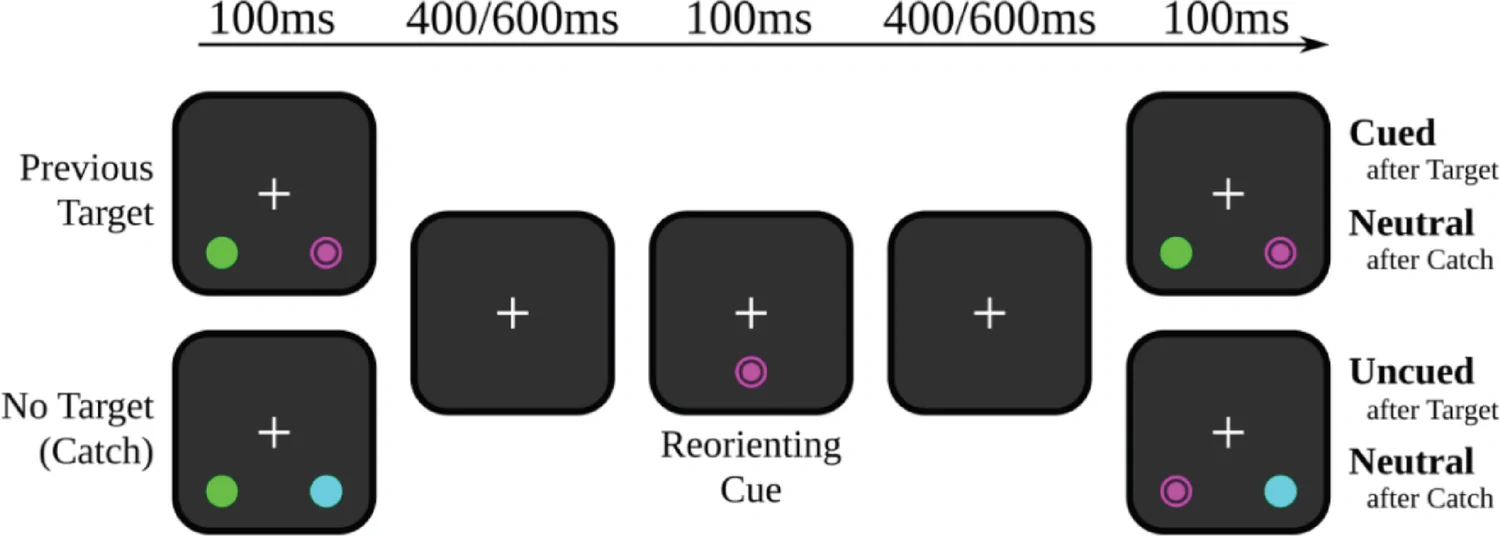
Schematic diagram of example trials from the inhibition of return task; the (pink) target is marked by an embossed edge for illustration purposes. In the first display, participants identify if the target is on the left or the right of the screen, followed by an attention re-orienting target flash. The next target then appears either in the same location as the previous target (cued trial) or the opposite location (uncued trial). Non-target disc colours varied randomly across trials

The IOR task was adapted from McDonald et al. (2008). It was programmed using PsychoPy software (Peirce 2007) and presented using a 17" LED monitor. Pairs of coloured discs were presented sequentially on a black screen, four degrees below and six degrees to the left and right of a central fixation cross, at a viewing distance of 55 cm. Participants were instructed to maintain fixation on the central cross throughout the task. Discs were either pink, green, or blue, and were visually matched in size and luminance. Following the presentation of disc pairs, a single target-coloured disc was presented beneath the fixation cross. This served to disengage the participants attention from a lateralised location (McDonald et al. 2008). See Fig. 1 for a schematic diagram depicting the timings of stimulus presentations.

Participants were assigned a target disc colour and were asked to identify if the target appeared on the left or right side of the screen using their index and middle finger of their dominant hand (left and right keys were *b* and *n* keys of a standard English keyboard, respectively). The target colour remained consistent during the visit, but was counterbalanced across study visits. The non-target distractor colours were randomly selected from the remaining two colours on each trial, ensuring that colour repetition and spatial repetition were not systematically confounded. Figure 1 illustrates one example sequence; in the full set of trials, distractor colours varied unpredictably across trials. Catch trials (no target present) comprised one-third of all trials (342 trials) and were randomly interspersed. Neutral trials (targets following catch trials) emerged from this randomisation, also comprising approximately 342 trials. The remaining target trials were equally split between cued (target on the same side as preceding target) and uncued (target on the opposing side to preceding target) conditions (171 each). Coloured discs were presented equally often on the left or the right side of the screen. After completing 22 practice trials, participants were presented with 1,026 trials in total, split into 27 equal blocks, each lasting ~ 45 s. Between blocks, participants took self-paced short breaks and were provided with a longer break halfway through the task. The task was completed in 20–30 min including explanation, practice, and breaks.

#### Electrophysiology

##### Event-related potentials

EEG was recorded using sixty-four *Easycap* scalp electrodes (easycap.de), at a sampling rate of 1000Hz, and amplified by a *Brainvision BrainAmp DC plus MR* amplifier. Before ERP extractions, EEG recordings were preprocessed off-line; electrodes were re-referenced to a whole-scalp reference, low-pass filtered at 40Hz, down sampled to 200Hz, and then high-pass filtered at 0.1Hz. ERP epochs were then defined as -100ms to 400ms relative to the onset of the target display, were averaged, and baseline-corrected relative to the 100ms pre-stimulus time window. Trials contaminated by artefacts (including eye-blinks/movements) were excluded from analyses, detected as events recorded at any of the electrode channels exceeding 75μV relative to the pre-stimulus baseline. Incorrect trials (and trials directly following an incorrect response) were excluded from analyses.

Average P1 and N2pc mean latencies and amplitudes were obtained for each participant in each condition (cued, uncued, and neutral) and for each target location (left and right). P1 was defined as the first positive peak of the signal occurring across posterior electrodes contralateral to the target location (electrodes P6/7, PO6/O7, P3/4, PO3/O4). N2pc was measured at the aforementioned posterior electrode sites as the peak difference between signals ipsilateral and contralateral to the target location, occurring in a time window of 160-260ms post stimulus onset. The mean amplitude values of P1 and N2pc were quantified as the average voltages 10ms and 20ms either side of the ERP peaks, respectively (Luck and Hillyard 1994). ERP latencies were measured as the time of peak ERP amplitude relative to the target onset.

##### Eye-blink rate

Using frontal EEG electrodes, individual blinks were quantified using independent component analysis of continuous EEG recordings down sampled to 200 Hz and filtered offline. Independent component analysis (ICA) was performed using the jader function from the EEGLAB open-source toolbox. The component corresponding to eye-blinks was identified via visual inspection. Blinks were quantified using a custom Matlab script to count the number of prominent voltage deflections in the identified component. Contrast analysis was employed to test for the predicted linear relationship between dopamine manipulation and EBR.

### Primary and exploratory analyses

Our primary hypotheses and planned analyses were:

1. Effects of drug condition (antagonist, placebo, agonist) on IOR magnitude (cued minus uncued RT difference).
2. Effects of drug condition on cued and uncued RTs separately.
3. Effects of drug condition on spontaneous eye-blink rate.
4. Effects of drug condition and cue condition on P1 and N2pc ERP amplitudes and latencies.
5. Main effects of cue condition (cued vs. uncued) on ERPs.

The following analyses were exploratory, planned after examining the primary results:

1. Correlation between individual differences in agonist effects on cued RTs and N2pc amplitudes: This analysis was motivated by the observation that both drug conditions reduced cued RTs, prompting investigation of whether individual differences in behavioral drug effects related to ERP changes.
2. Analysis of neutral trial RTs: This analysis was conducted to verify that drugs did not affect baseline response speed, which would confound interpretation of the IOR effects.
3. Speed-accuracy and proportional IOR analyses (Supplementary Materials): Conducted to rule out artifacts of speed-accuracy trade-offs and general response slowing.

## Results

One participant experienced an adverse reaction to amisulpride and consequently withdrew before completing the study. Another participant withdrew before completing all three sessions. Therefore, the remaining data from these two participants were not included in analyses.

### Eye-blink rate

**Fig. 2:**
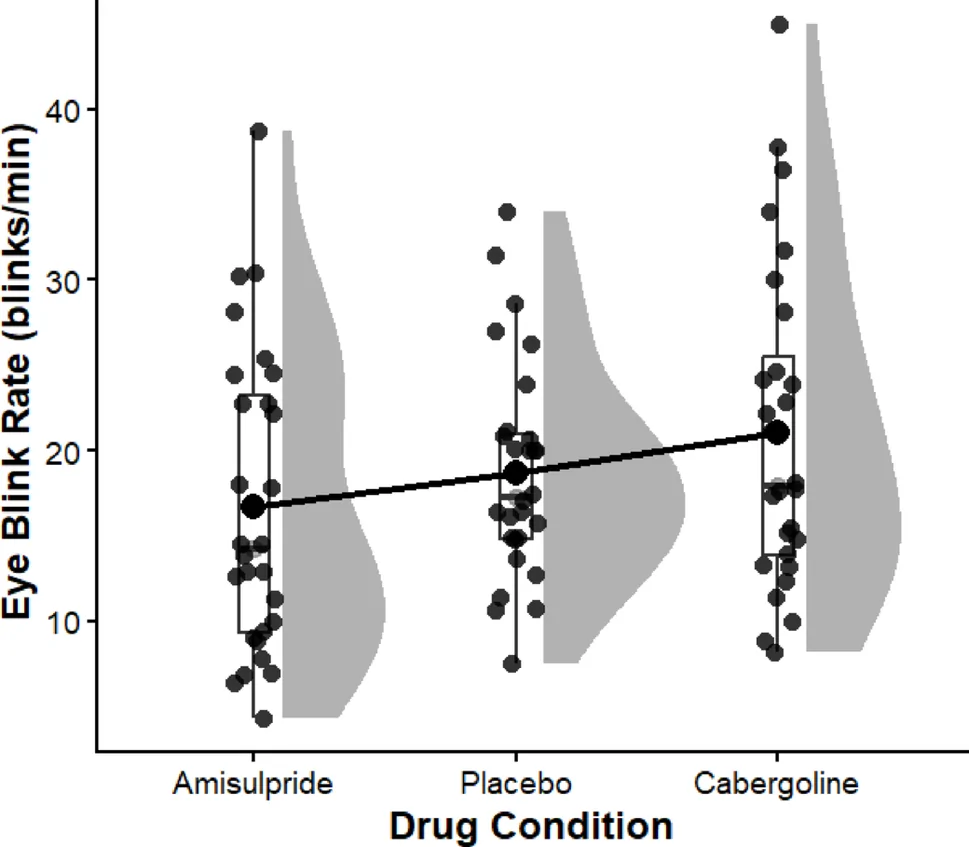
Distribution of eye blink rate (EBR) by drug condition. Individual data points are overlaid on kernel density estimates (cloud) and box plots (median and interquartile range). Line connects group means. Striatal dopamine reduction using a D2 antagonist (amisulpride) reduced spontaneous eye-blink rates, whereas a dopamine agonist (cabergoline) increased EBRs, relative to placebo

One participant showed an EBR rate more than 1.5 times higher than the interquartile range for the antagonist group and was therefore removed from the EBR analysis. There was a significant linear relationship between dopamine level and EBR; lowest, medium, and highest EBR rates were found in the antagonist, placebo, and agonist conditions, respectively; F(1, 27) = 7.242, *p* = 0.012; see Fig. 2. There was no significant nonlinear relationship between EBR and dopamine manipulation (*p* = 0.926). This is consistent with the hypothesis that the dosages of amisulpride and cabergoline administered in this study achieved a decrease and increase in striatal dopamine signalling, respectively, due to their action on postsynaptic rather than presynaptic D2 autoreceptors (Frank and O’Reilly, 2006).

### Behaviour

One participant’s placebo IOR scores were not saved due to computer error. Therefore, their data were not included in further analyses. The remaining twenty-eight participants had a mean incorrect response rate of 3.7% (SD = 1.5). Outlier analysis using 1.5 times IQR criterion confirmed that no participant (including the two left-handed participants) exceeded outlier thresholds in any condition. RTs underwent an outlier removal procedure (cf. VanSelst and Jolicoeur 1994), which removed an average of 2.2% (SD = 0.7) of trials from each participants responses in each visit. All three drug conditions produced slower responses to the cued versus the uncued location.

Using a 2 × 3 ANOVA, there was a significant main effect of drug on IOR reaction times (cue condition); F(2,54) = 5.92, *p* = 0.005, ηp2 = 0.180. Tests of simple effects demonstrate that both the agonist (M = 344ms; t(27) = 3.30, *p* = 0.003, *d* = 0.624) and antagonist (M = 342ms; t(27) = 2.65, *p* = 0.013, *d* = 0.501) significantly decreased RTs compared to placebo (M = 354ms). There was no significant difference between agonist and antagonist RTs (*p* = 0.644). The main effect of cue condition was significant; F(1, 27) = 27.70, *p* < 0.001, ηp2 = 0.506. Tests of simple effects revealed that across dopamine manipulations, cued RTs (M = 356ms) were significantly slower than uncued RTs (M = 337ms); t(27) = 5.26, *p* < 0.001, *d* = 0.994 (i.e. the IOR effect).

**Fig. 3:**
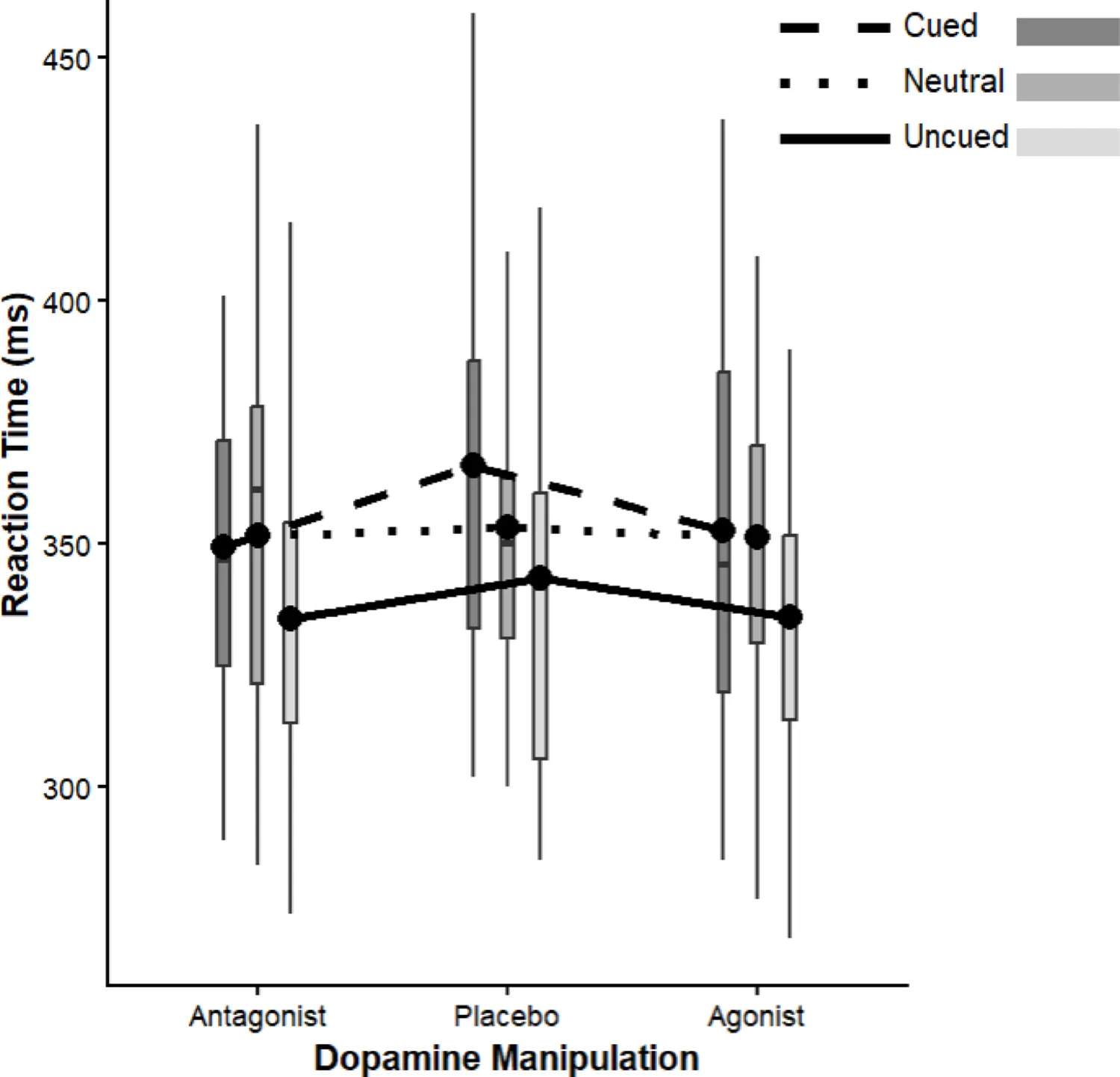
Reaction times by drug condition and trial type. Box plots show the distribution of individual participant reaction times (median and interquartile range). Black points represent mean reaction times connected by lines. Error bars represent within-subject standard error. Changes in striatal dopamine (increase and decrease with D2 agonist and antagonist, respectively) reduce cued reaction times. Neutral trials do not differ between conditions. Individual RT distributions for all conditions are shown in Supplementary Figure S1

The interaction between dopamine manipulation and cue condition was significant; F(2, 54) = 6.29, *p* = 0.004, ηp2 = 0.189. Planned tests of simple effects determined that cued RTs were significantly faster in the agonist (M = 353; t(27) = 3.94, *p* = 0.001, *d* = 0.745) and antagonist (M = 349; t(27) = 3.33, *p* = 0.003, *d* = 0.629) manipulations compared to placebo (M = 366ms). There were no significant differences in uncued RTs between dopamine manipulations after Bonferroni correction for three pairwise comparisons (threshold p < 0.017): Agonist vs. placebo, *p* = 0.025; antagonist vs. placebo, *p* = 0.090; agonist vs. antagonist, p = 0.950. See Fig. 3.

Cued minus uncued RTs provided a measure of IOR magnitude. A significant quadratic contrast was observed, consistent with a non-linear relationship between IOR magnitude and drug condition; F(1, 27) = 8.59, *p* = 0.007, ηp2 = 0.241. There was no linear relationship between variables (*p* = 0.138). To investigate the nonlinear effect of dopamine manipulation on IOR magnitude, a one-way ANOVA was utilised with three levels: antagonist, agonist, and placebo. The main effect was significant; F(2,54) = 6.29, *p* = 0.003, ηp2 = 0.189. T-tests of simple effects identified that the dopamine agonist (M = 17.9); t(27) = 2.37, *p* = 0.025, *d* = 0.448; and antagonist (M = 14.9); t(27) = 2.96, *p* = 0.006, *d* = 0.559; both showed smaller IOR magnitudes compared to placebo (M = 22.9). Therefore, results are consistent with a non-linear relationship between dopamine level and IOR magnitude. To ensure these effects were not artifacts of general response slowing or speed-accuracy trade-offs, we conducted two supplementary analyses (Supplementary Materials S1.2 and S1.3). Both support the pattern of results: accuracy rates did not differ between conditions, and proportional IOR scores. replicated the drug effects, demonstrating that the drugs specifically reduced spatial attentional inhibition rather than affecting response speed generally.

A one-way ANOVA was performed to test the effect of dopamine manipulation on mean RTs in neutral trials. This was to establish whether there was a significant shift in participant’s baseline RTs which should be accounted for when interpreting the above findings. However, neutral RTs did not differ between placebo (M = 353, SD = 35), agonist (M = 351, SD = 37), and antagonist (M = 352, SD = 37); F(2,54) = 0.107, *p* = 0.899, ηp2 = 0.004.

### Electrophysiology

**Fig. 4:**
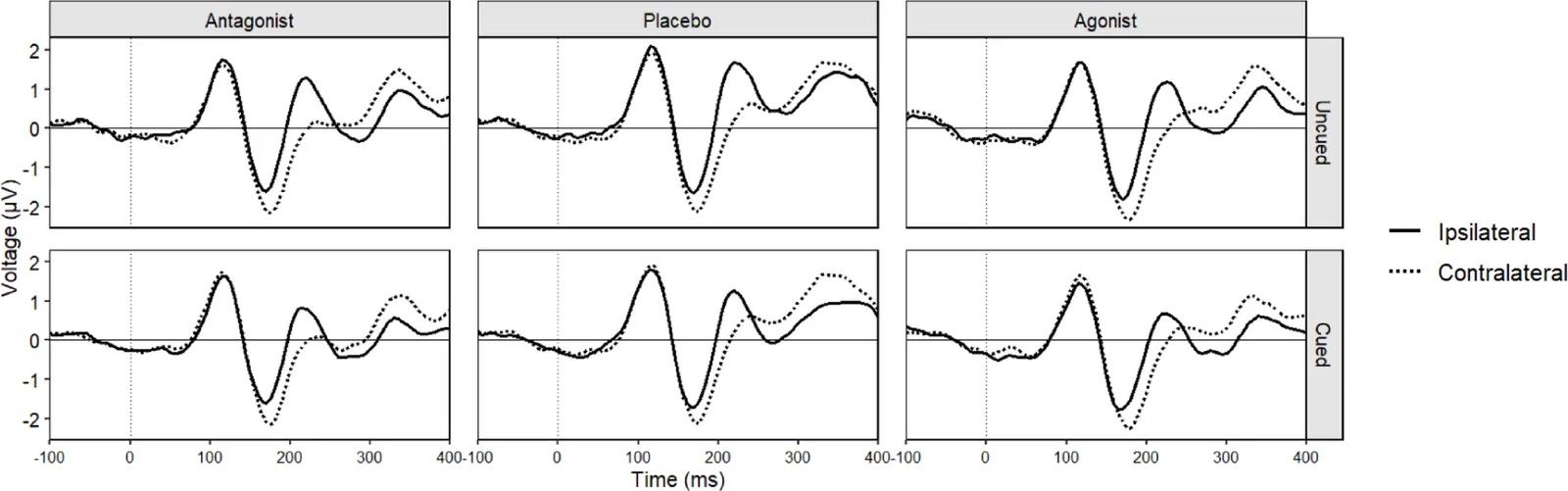
Event-related potential (ERP) waveforms elicited by target displays of the inhibition of return (IOR) task. Signals were averaged from posterior electrode sites (P6/7, PO6/O7, P3/4, PO3/O4) contralateral and ipsilateral to the target location for each dopamine manipulation and cue condition. No single ERP shows significant differences between dopamine manipulations

**Fig. 5:**
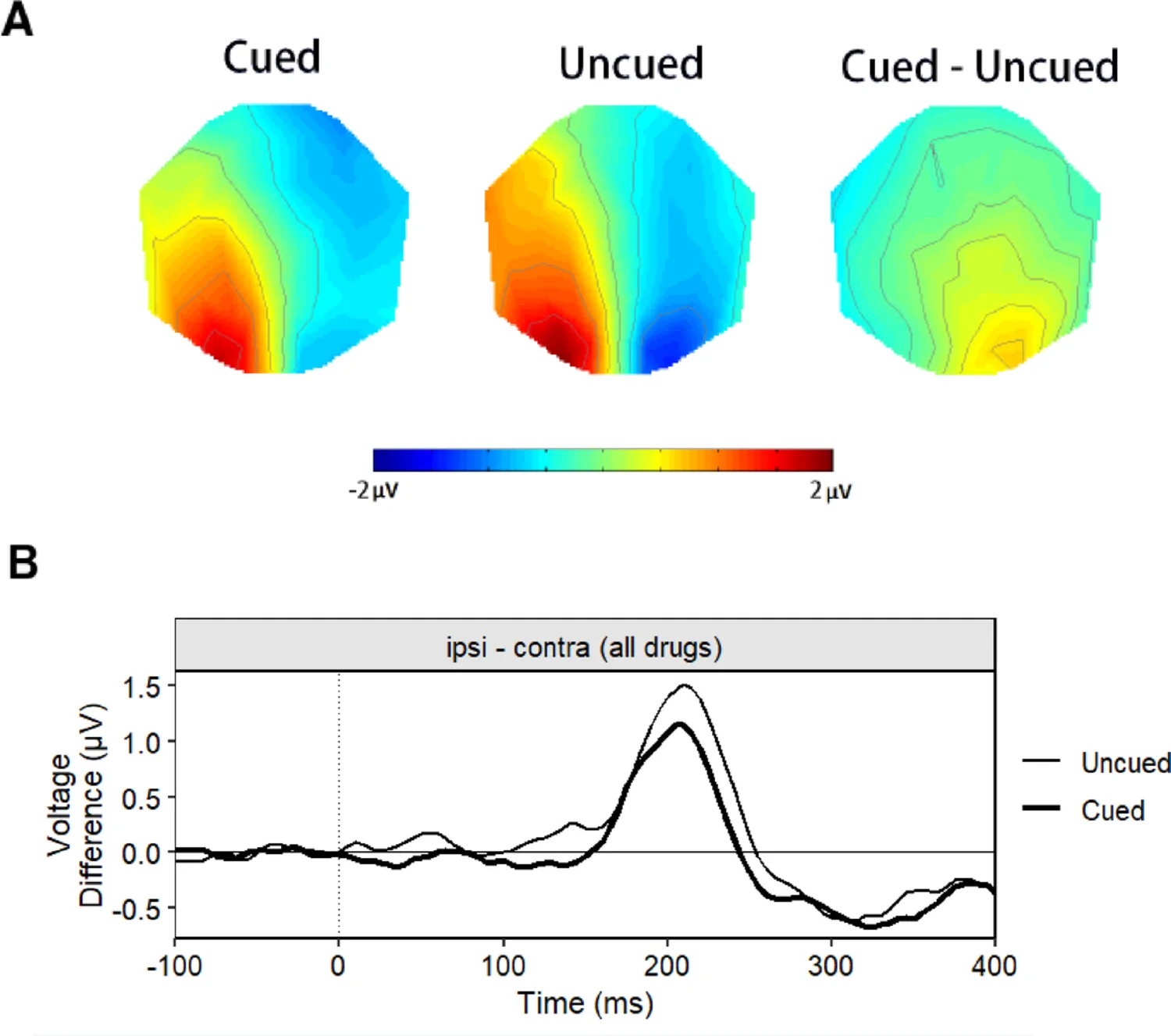
The N2pc was significantly larger in uncued versus cued IOR trials.** A** Topographical maps of the N2pc, obtained from left-side minus right-side target locations for cued and uncued trials. **B** ERP plots of N2pc difference waves derived from electrodes ipsilateral minus contralateral to the target

**Fig. 6:**
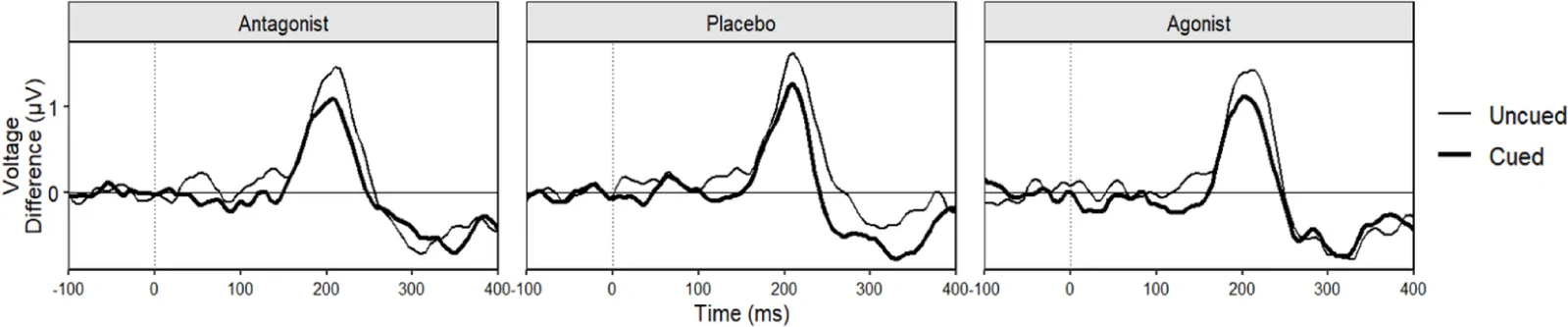
Cued and uncued N2pc difference waves (ipsilateral minus contralateral) for each drug condition. There was no main effect of drug or interaction between drug and cue conditions

The data from three more participants were omitted from ERP analyses (as well as the aforementioned three participants). This was due to the lack of discernible visual-evoked potential in averaged waveforms, defined as a prominent positive peak at posterior electrodes between 70-140ms following stimulus presentation (cf. Di Russo et al. 2002). For the remaining twenty-five participants, see Fig. 4 for ERPs obtained for each task condition. No cued minus uncued ERP latency or amplitude showed significant quadratic contrast (non-linear) relationships with dopamine manipulation (see Table 2). See Table 3 for results of two-way ANOVAs; the significant main effect of cue condition on N2pc amplitude was further explored using pairwise comparisons. N2pc amplitude was significantly larger in the uncued cue condition (M = 1.63µV) compared to the cued condition (M = 1.30µV); t(24) = 2.86, *p* = 0.009, *d* = 0.540; see Fig. 5. This implies that selective attention was stronger for uncued targets compared to cued targets. No other main effects or interactions reached statistical significance (see Fig. 6 for ERPs). These null ANOVA results may indicate that the effects of the drugs on IOR may be different for cued versus uncued ERPs, or involve more than a single ERP latency/amplitude.

**Table 2:** Contrast analyses of the relationship between dopamine manipulation and event-related potentials (ERPs)

ERP		Contrast	F	*P*
N2pc	Amplitude	Linear	0.709	0.408
		Quadratic	0.086	0.771
N2pc	Latency	Linear	0.002	0.964
		Quadratic	0.069	0.795
P1	Amplitude	Linear	0.058	0.812
		Quadratic	0.232	0.634
P1	Latency	Linear	2.667	0.116
		Quadratic	0.540	0.469
Pd	Amplitude	Linear	1.191	0.286
		Quadratic	1.347	0.257

degrees of freedom = 1,24

**Table 3:** ANOVA results analysing the effect of dopamine drug condition cue condition (cued, uncued) on event related potential (ERP) latencies and amplitudes

ERP		Effect	*c!f*	F	η_p_^2^	*p*
N2pc	Amplitude	Drug	2,48	0.234	0.010	0.792
		Condition	1, 24	8.162	0.254	0.009*
		Drug X condition	2,48	0.377	0.015	0.688
N2pc	Latency	Drug	2,48	1.180	0.047	0.316
		Condition	1, 24	0.923	0.037	0.346
		Drug X condition	2,48	0.032	0.001	0.968
Pl	Amplitude	Drug	2,48	0.700	0.028	0.502
		Condition	1, 24	0.107	0.004	0.747
		Drug x condition	2,48	0.142	0.006	0.868
Pl	Latency	Drug	2,48	0.953	0.040	0.393
		Condition	1, 24	0.162	0.007	0.691
		Drug X condition	2,48	1.647	0.067	0.204
Pd	Amplitude	Drug	2,48	0.207	0.009	0.814
		Condition	1, 24	0.158	0.007	0.695
		Drug X condition	2,48	1.257	0.050	0.294

*df* = degrees of freedom, *p* = * statistical significance < .05, η_p_^2^ = partial eta squared measure of effect size

Further exploratory analyses were guided by behavioural findings, using linear regression analyses to find which ERP(s) best related to notable behavioural effects. Cued and uncued trials were treated separately to account for potential differences in their processing.

As behavioural results demonstrated that the reduction in IOR magnitude were driven by reductions in cued RTs for both drugs, further analyses focused on cued responses; the *agonist effect* was quantified as mean cued placebo RTs minus cued agonist RTs for each participant. The *antagonist effect* was quantified as mean cued placebo RTs minus cued antagonist RTs for each participant. Agonist and antagonist effects were also quantified in the same manner for each ERPs latencies and amplitudes.

One participant showed a behavioural antagonist effect more than 1.5 times lower than the interquartile range, and therefore their data were not included in analyses of the antagonist effect. No outliers were identified for the behavioural agonist effect.

**Fig. 7:**
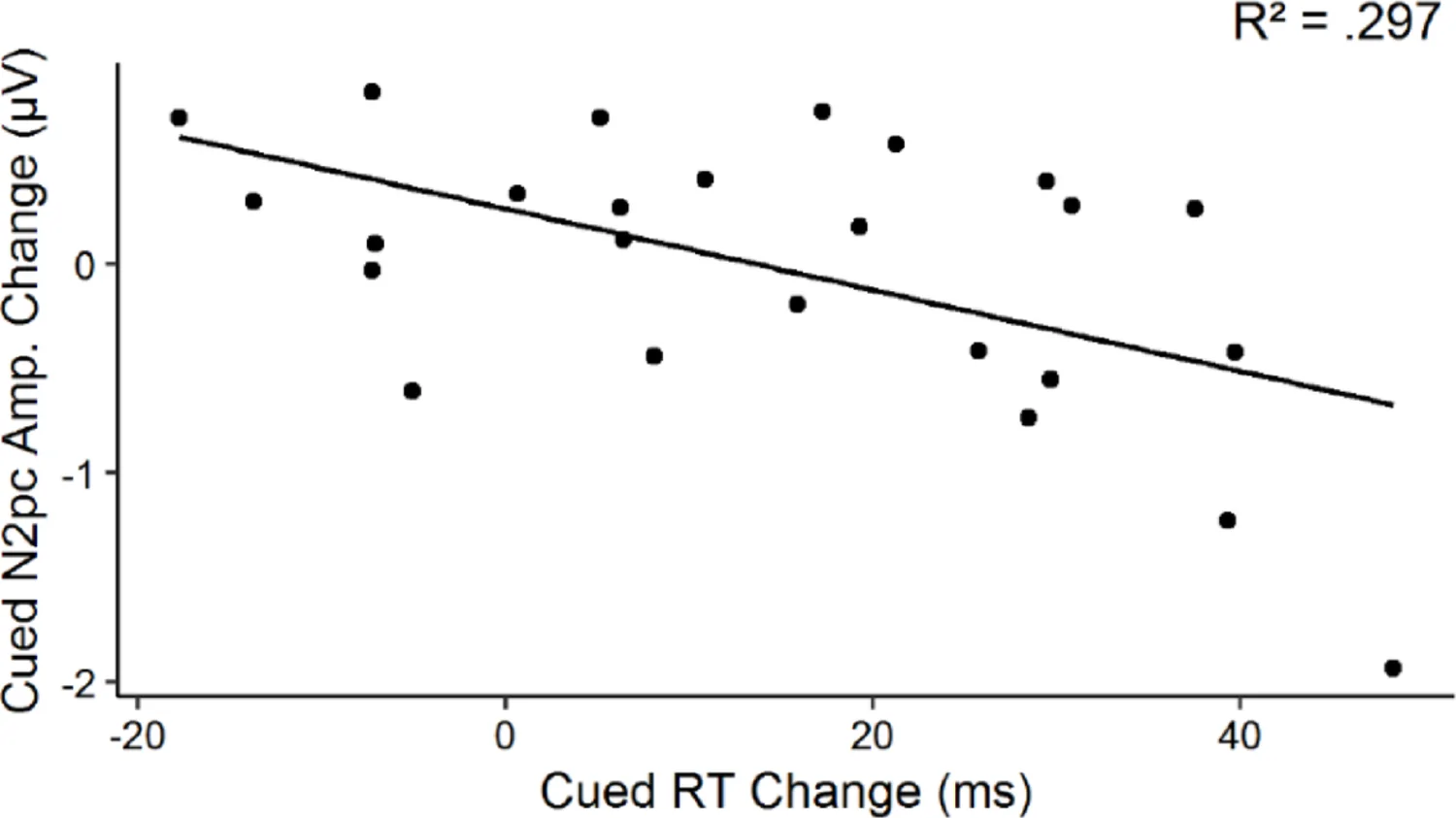
Faster reaction times to cued targets were related to larger N2pc amplitudes following D2 agonist administration, indicating increased selective attention to cued locations

The behavioural agonist effect was related to changes in N2pc amplitude; F(1, 24) = 9.709, *p* = 0.005, R^2^ = 0.297. All other ERPs were excluded from the model. This indicated that faster cued RTs in the agonist condition were related to larger N2pc amplitudes relative to placebo (see Fig. 7). No ERPs were significant predictors of the antagonist effect. As noted above, this correlation analysis was exploratory and should be interpreted with appropriate caution (see “Mechanistic insights from ERPs” section).

## Discussion

In this study, we investigated the role of striatal dopamine in the attentional phenomenon of IOR. To achieve this, dopamine signalling was pharmacologically altered at D2Rs, and the subsequent effects on reaction times and cognitive processes (ERPs) were measured. Eye-blink rate (EBR) was used to monitor the effects of the drugs. EBR was highest in the D2R agonist condition and lowest in the D2R antagonist condition, consistent with the intended dopaminergic manipulations. While the interpretation of EBR as a dopamine proxy has important limitations (discussed in “Eye-blink rate as an index of dopaminergic effects” section), this directional pattern supports that the drugs had central dopaminergic effects, allowing us to interpret the subsequent behavioral and electrophysiological findings in the context of altered dopaminergic signaling.

### Eye-blink rate as an index of dopaminergic effects

Dopamine agonists consistently increase blink rate while antagonists decrease it (Blin et al. 1990; Depue et al. 1994; Kleven and Koek 1996), and clinical populations with dopamine dysfunction show altered EBR that responds to dopaminergic treatment (Jongkees and Colzato 2016). The pattern observed in this study (highest EBR with cabergoline, lowest with amisulpride) aligns with this evidence, consistent with our drugs having central dopaminergic effects.

However, Positron Emission Tomography (PET) imaging studies have failed to find correlations between EBR and dopamine measures in healthy adults (Dang et al. 2017; Sescousse et al. 2018; van den Bosch et al. 2023), challenging the assumption that EBR reliably indexes striatal dopamine in healthy populations.

This discrepancy may reflect methodological differences: Jongkees and Colzato (2016) suggest that EBR may index dynamic dopamine release capacity rather than static receptor density or baseline synthesis, which would explain why acute pharmacological manipulations produce reliable EBR changes while correlations with static PET measures are inconsistent. The EBR-dopamine relationship may also be non-linear or only evident at extremes of function, as stronger associations are observed in clinical populations with dopamine dysfunction than in healthy volunteers (Jongkees and Colzato 2016).

Nevertheless, two key uncertainties limit precise mechanistic interpretation of EBR effects in this study. First, dose considerations: Naef et al. (2017) demonstrated significant cognitive impairments from D2 antagonism only at 800mg sulpiride versus our 400mg amisulpride dose, raising the possibility of mixed pre- and post-synaptic actions. Second, anatomical localisation: effects likely involved both striatal and cortical regions. While we have framed our findings primarily in terms of striatal dopamine, our D2R-selective drugs act on both striatal and cortical D2Rs (Durstewitz and Seamans 2008), and behavioral effects likely reflect combined frontostriatal contributions.

Despite these uncertainties, the directional consistency with established pharmacological evidence supports interpreting our findings in the context of altered dopaminergic signaling, though the mechanistic interpretation of behavioral effects should be considered within these limitations.

### Behavioural findings

Both the D2 agonist and antagonist reduced the IOR effect, relative to placebo. This is consistent with a non-linear relationship between striatal dopamine and IOR, with intermediate levels producing greatest effects. Furthermore, we provide evidence consistent with the involvement of striatal dopamine signalling by using D2R-selective drugs (cabergoline and amisulpride) (Correll et al. 2004; Odin et al. 2006). This pattern aligns with previous findings that dopaminergic dysfunction (hyper- and hypo-) reduces IOR (see Table 1, Introduction), though moderate increases from low baseline can enhance IOR (Rokem et al. 2012).

The present study extends previous findings by showing the direct effects of reduced dopamine signalling on IOR, thus demonstrating three points along a non-linear function within the same participant group. Furthermore, we analysed the effects of drugs on cued and uncued trials separately and found that both the D2 agonist and antagonist reduced cued RTs compared to placebo.

There was no statistically significant effect of the drugs on uncued RTs. However, it should be noted that the effect of the agonist on uncued RTs approached statistical significance. Indeed, it is possible that attending towards novel locations might have been faster in the case of uncued RTs, but effects may have been limited by human response speeds (i.e. floor effect) (Liesefeld and Janczyk 2019). Consequently, it is difficult to conclude if excessive or insufficient striatal dopamine may solely reduce *inhibition* of previously attended visual locations, or speed the cued and uncued RTs alike.

The marginal trend toward faster uncued RTs in the agonist condition (p = 0.025, uncorrected) is noteworthy, despite not reaching statistical significance after Bonferroni correction. One possible interpretation is that the drugs did not globally degrade performance but may have redistributed attentional resources: the agonist may have both reduced inhibition at cued locations (speeding cued RTs) and enhanced vigilance for uncued targets (marginally speeding uncued RTs). This redistribution between tagging previously attended locations versus monitoring for novel stimuli would be consistent with the stability-flexibility trade-off discussed above. However, given that this effect did not survive correction for multiple comparisons, this interpretation remains tentative and requires replication.

It is important to acknowledge that with only three data points (antagonist, placebo, agonist), we cannot definitively characterise the precise form of the non-linear relationship between dopamine and IOR. As Floresco et al. (2013) noted, the optimal balance between D1R and D2R stimulation can produce various non-linear functions depending on the task, including quadratic, cubic, or other forms. Our data are consistent with a non-linear relationship (including, but not limited to, an inverted-U form). Additional intermediate doses would be required to fully characterise the shape of this dose–response curve and determine whether the relationship is truly an inverted-U or follows another non-linear pattern.

### Attentional vs. response-level mechanisms

An important consideration in interpreting our findings is whether the observed effects reflect spatial attentional inhibition (as traditionally conceived in IOR) or response-level processes (cf. Poliakoff et al. 2007). One possibility is that making a response to a location creates post-response motor inhibition that slows subsequent responses to that same location, independent of spatial attention.

Several lines of evidence argue against this as the primary mechanism. First, our neutral trial data provide direct evidence that drugs did not affect motor response speed per se. Neutral trials (following catch trials) require motor responses but lack the spatial repetition/novelty manipulation that defines cued versus uncued conditions. The absence of drug effects on neutral trial RTs (p = 0.899) indicates that the drugs did not affect baseline motor speed. If drugs affected post-response motor inhibition mechanisms generally, we would expect effects on neutral trial RTs as well. Second, we employed a central attention-reorienting flash between disc pairs (McDonald et al. 2008) to disengage attention from lateral locations and dissipate lingering motor activation. Thirdly, Prime and Ward (2006) demonstrated IOR effects without corresponding lateralised readiness potential (LRP) modulation in a cue-target paradigm. Although no LRP analyses were performed in the present study, Prime and Ward's null LRP findings, combined with our neutral trial data showing no drug effects on baseline motor speed, suggest motor-level delays are unlikely to fully account for our effects.

Nevertheless, our task design does not permit complete isolation of attentional from motor processes. As noted in our Methods, cued trials involve both spatial and response repetition, while uncued trials involve both spatial and response switching. Although non-target disc colours varied randomly across trials (preventing systematic confounding of colour with spatial repetition), the inherent coupling of spatial location with response direction means our measured IOR effect could reflect spatial attentional inhibition, response switching costs, or a combination. This feature is characteristic of spatial IOR paradigms generally, (Klein 2000; Lupiáñez et al. 2006) rather than a limitation unique to our design.

We therefore interpret IOR and its modulation by dopamine as involving multiple mechanisms: spatial attentional tagging, maintenance of priority maps, and potentially sequential motor effects, with our findings most consistent with dopamine affecting attentional/cognitive rather than purely motor processes. Supplementary analyses support this interpretation: proportional IOR scores replicated the pattern of drug effects, and accuracy rates did not differ between conditions, ruling out speed-accuracy trade-offs (Supplementary Materials S1.2-S1.3). Future research using tasks that fully dissociate spatial attention from response selection (e.g., orthogonal cue-response mappings) would provide stronger evidence for the specific locus of dopaminergic effects on IOR.

### Theoretical framework

To understand how both excessive and insufficient dopamine could produce similar behavioral effects, we consider the underlying neurobiological mechanisms that may be involved. Dopamine is proposed to have different, even competitive, roles in the striatum versus the prefrontal cortex (Cools and D'Esposito 2011); prefrontal cortex stimulation via D1Rs stabilises attention and provides resistance to distraction, whereas D2R stimulation encourages cognitive flexibility (Durstewitz et al. 2000; Holstein et al. 2011). A study by Riedel et al. (2022) administered L-DOPA, demonstrating a causal influence of dopamine levels on the stability-flexibility trade-off in attentional control during visual search. Other studies have shown that dopamine in the prefrontal cortex plays a crucial role in cognitive control by modulating the motivation to exert control processes (Ott and Nieder 2019; Cools et al. 2019). The relative balance of prefrontal cortex versus striatal dopamine signaling is mediated by reciprocal frontostriatal connections (Alexander et al. 1986; Morris et al. 2016), allowing individuals to both maintain stable task goals whilst preserving the ability to shift attention and update strategies (Cools and D'Esposito 2011; Cools et al. 2008).

The structural and functional integrity of frontostriatal circuits is crucial for optimal dopaminergic function. Developmental studies show that compulsivity and impulsive traits are linked to attenuated frontostriatal myelination trajectories (Ziegler et al. 2019), while prefrontal and frontostriatal structural variations mediate academic outcomes associated with ADHD symptoms (Chiu et al. 2021). These findings suggest that the circuit-level effects we propose for IOR depend not only on neurochemical balance but also on the structural integrity of frontostriatal pathways. Recent evidence suggests that this balance is particularly important for the subjective effort costs associated with cognitive control, with dopaminergic medications reducing these costs and increasing motivation to exert control (Bogdanov et al. 2022).

IOR is thought to involve the application of an “inhibitory tag” to previously cued locations, following attentional disengagement from that location (Tian et al. 2011). This demarks the location as “already inspected” and therefore not to be prioritised by the attention system (Klein 2000; Lupiáñez et al.; 2006). IOR may be considered a mechanism of cognitive flexibility, as it promotes the inspection of novel stimuli in the environment (Colzato et al. 2010; Klein 2000). Attentional disengagement is thought to be key for the IOR effect (Klein 2000; Tian et al. 2011), and a lack of disengagement may result in more facilitation effects of the cued location (Colzato et al. 2010). One possibility is that decreased striatal dopamine signalling via the D2R antagonist *amisulpride* may reduce IOR by biasing the frontostriatal system away from flexibility. A more stable system may have held attention at cued locations, which in turn could prevent or delays the formation of the inhibitory tag necessary to produce IOR.

Alternatively, a reduction in striatal signalling may negatively impact inhibitory tag formation, as striatal dopamine signals are thought to be important for the SC to produce such tags (Ikemoto et al. 2015; Klein 2000). Evidence suggests that the deeper layers of the SC are crucial for attentional modulation and contain specialised functional modules for spatial orienting (Masullo et al. 2019; Ding et al. 2019). The absence of appropriate dopaminergic input from the BG to these specialised SC circuits could bias the attention system toward external sensory cues, potentially causing the sensory element of the stimulus to override IOR. This is in accordance with the general hyper-reflexivity observed in patients with Parkinson's disease (Jackson and Houghton 1995; Briand et al. 2001; Kingstoneet al. 2002), along with their attenuated spatial and tactile IOR effect (Poliakoff et al. 2003; Possin et al. 2009).

Conversely, an excessive increase in striatal dopamine signalling caused by cabergoline may promote an overly-flexible state that could interfere with prefrontal cortex activity (Meyer-Lindenberg et al. 2005). The prefrontal cortex may then be less able to maintain inhibitory tags, which could contribute to faster cued RTs. This interpretation is tentatively supported by the exploratory correlation between faster cued RTs and larger N2pc amplitudes in the agonist condition (discussed in “Mechanistic insights from ERPs” section), though this finding requires replication.

The present findings align with evidence that dopamine's effects on cognitive control follow a non-linear function, where both too little and too much dopamine can impair performance (Badre 2025; Friedman and Robbins 2022). Research has shown that dopaminergic medications can improve cognitive control under low cognitive demand conditions (Williams et al. 2020), but effects may depend on baseline dopamine levels and individual differences (Furman et al. 2020). Our results suggest that IOR, as a form of attentional control, may be subject to similar dopaminergic modulation. The non-linear relationship we observed between striatal dopamine and IOR magnitude may reflect the optimal balance needed for effective cognitive control processes, where intermediate dopamine levels support the most efficient attentional inhibition. This perspective is further supported by research demonstrating that dopamine plays a multi-faceted role in working memory, attentional control, and intelligence through dual-state signaling mechanisms (Matzel and Sauce 2023). However, as noted in our behavioral findings, our three-dose design limits our ability to specify the exact functional form of this relationship, and future research with additional intermediate doses would help determine whether IOR follows the same inverted-U pattern observed in working memory or a different non-linear function.

In summary, we propose that the drugs cabergoline and amisulpride shifted the balance between responding to novel targets (attentional flexibility) versus maintaining inhibitory tags from previous locations (attentional stability), though the precise mechanisms remain to be established. The reduced IOR under both drug conditions could reflect interference with inhibitory tag formation or maintenance via effects on the SC, prefrontal cortex, or their interactions, but our data cannot distinguish among these possibilities. Future research should test these mechanistic hypotheses more directly by: (1) manipulating dopamine specifically in prefrontal cortex versus striatum to isolate their contributions, (2) using neuroimaging to measure frontostriatal connectivity changes under dopaminergic manipulation, and (3) establishing how ERP modulations relate to inhibitory tag formation and the proposed frontostriatal balance in IOR. Such work would clarify whether the theoretical framework outlined here accurately captures the mechanisms underlying dopaminergic modulation of IOR.

### Mechanistic insights from ERPs

While the behavioral findings support this theoretical framework, we also examined electrophysiological measures to gain insight into the underlying neural mechanisms. We found that N2pc amplitudes were greater for uncued versus cued targets, irrespective of dopamine manipulation, showing typical attentional effects on ERPs alongside the behavioural measures (McDonald et al. 2008).

Striatal dopamine manipulations did not significantly affect the difference between cued and uncued N2pc amplitudes or latencies, or that of P1. This indicates that the drugs may have affected cued and uncued target processing differently, and/or affected multiple stages of processing in IOR. Therefore, to account for such complexity, we investigated the potential relationships between how drugs affected RTs and ERPs by analysing their relationship separately for cued and uncued conditions. We found that faster RTs to cued targets in the D2R agonist condition were related to greater N2pc amplitudes. In other words, increased striatal dopamine was related to increased attention to cued targets (Kiss et al. 2008). However, this correlation should be interpreted cautiously. The analysis was exploratory, arising from post-hoc examination of individual differences rather than an a priori hypothesis. Given that we examined one specific drug × cue condition out of several possible combinations, and could have tested relationships with multiple ERP measures, the possibility of Type I error should be considered. This finding is best viewed as hypothesis-generating and would benefit from replication in an independent sample before drawing strong theoretical conclusions.

If supported by subsequent research, this pattern would align with more recent evidence that enhanced selective attention processes in IOR can manifest as increased N2pc amplitudes even when behavioral response times are faster (Lin et al. 2020), suggesting that dopamine agonism may optimise the efficiency rather than simply the speed of attentional selection. Such a pattern would suggest that the D2R agonist reduced inhibition of cued targets, allowing attention to be captured more strongly – thus speeding RTs and reducing overall IOR.

There were no ERP correlates of the behavioural D2R antagonist effect. This could be attributed to several factors, including potential limitations in statistical power for detecting subtle ERP modulations. Other decision-making or motoric processes may have contributed to the behavioural effects that were outside of the measures taken in the present study. Furthermore, the drug may have subtle effects on several processing stages, rather than larger effects on certain stages—making identification of effects more difficult.

In contrast to the null group-level effects, our use of correlational analyses between behavioral and electrophysiological drug effects proved more sensitive than traditional group-level comparisons, revealing that dopamine agonism specifically enhanced the relationship between selective attention (N2pc) and behavioral performance. This approach may be particularly valuable for pharmacological studies where drug effects on different ERP components may vary in magnitude and direction.

In summary, these findings provide some mechanistic insight into the primary behavioural results. The exploratory correlation suggests that the agonist effect may be related to changes in selective attention, whereas the antagonist effect may have influenced other aspects of cognition not captured by our ERP measures.

### Broader implications

The present findings contribute to emerging research on individual differences in IOR. Bielas et al. (2021) demonstrated that the level of ‘emotional reactivity’ in human temperament can differentially affect IOR magnitude, suggesting that the dopaminergic effects we observed may interact with personality-based differences in attention control.

Our results align with theoretical frameworks that position IOR as a sophisticated information processing mechanism rather than a simple inhibitory effect (Redden et al. 2021; Tsotsos 2021), with implications extending beyond basic attention research to understanding naturalistic visual behavior and clinical applications.

Previous research has shown that striatal dopamine is important for inhibitory processes (Cools and D'Esposito 2011), including response inhibition (Bari and Robbins 2013; Eagle et al. 2008). While correlational evidence suggests striatal involvement in IOR (Poliakoff et al. 2003; Colzato and Hommel 2009), the present study is the first to directly and pharmacologically manipulate striatal dopamine to examine its causal role in this rapid, reflexive attentional mechanism.

Furthermore, IOR is proposed to improve visual search efficiency by reducing unnecessary re-inspections of stimuli (Klein 2000; Li et al. 2023; Klein et al. 2023). If this is the case, the effects of dopamine manipulations on IOR might extend to higher processes such as visual search, though this remains to be tested. It would be valuable for future research to examine whether the dopaminergic modulation of IOR we observed translates to similar effects on visual search performance in real-world contexts.

Understanding the specific roles of dopamine signaling in attention may eventually inform the treatment of attentional disorders. Recent advances in D2R ligands for neuropsychiatric disorders have emphasised the need for more precise targeting of dopaminergic systems (Juza et al. 2023), while research on dopamine receptor expression patterns in ADHD has highlighted the complexity of dopaminergic dysfunction across different attentional disorders (Dum et al. 2022). Our findings raise the possibility that optimal therapeutic approaches may need to consider individual baseline dopamine levels to avoid both under- and over-stimulation of D2R systems, though translating these findings from healthy volunteers to clinical populations requires further investigation.

Recent research has demonstrated that dopaminergic medications can selectively improve cognitive control processes in Parkinson's disease (Cavanagh et al. 2022), and that these effects may depend on the specific cognitive demands of the task (Williams et al. 2020). Multimodal investigations have also revealed complex relationships between dopamine D2/D3Rs, default mode network suppression, and cognitive control in disorders such as cocaine-use disorder (Worhunsky et al. 2021). For instance, dopamine-increasing treatments for Parkinson’s disease have unwanted, debilitating effects on attention and impulsivity (Weintraub et al. 2010), which may result from exceeding optimal dopamine levels for cognitive control (Bogdanov et al. 2022). This is thought to be caused by non-selective effects of dopamine drugs on different brain systems, as well as individual differences in patients' dopamine systems (Napier et al. 2015; Weintraub et al. 2006).

The present findings also highlight the need to consider the role of the SC in IOR within the broader context of attention networks. Recent work demonstrating distinct contributions of frontal eye field and SC to covert spatial attention (Bollimunta et al. 2018) suggests that dopaminergic manipulations may differentially affect these parallel attention systems. Given that the SC represents an evolutionarily conserved structure with both general and specialised functions for orienting behavior (Allen et al. 2021; Guillamón-Vivancos et al. 2024), understanding how dopamine modulates SC-mediated IOR may provide insights into fundamental mechanisms of attention that extend beyond human cognition.

Our findings may have implications for understanding attentional disorders across development, though this requires careful consideration. Research has shown that frontostriatal dysfunction characterises decision-making deficits in both ADHD and obsessive–compulsive disorder (Norman et al. 2018), while dopamine receptor expression patterns vary across different attention-deficit disorders (Dum et al. 2022). The non-linear relationship we identified raises the possibility that therapeutic interventions for these conditions may need to be carefully titrated to avoid pushing patients from one extreme (too little dopamine) to the other (too much dopamine), though whether our findings generalise to clinical populations remains to be established. Future research should investigate whether similar non-linear relationships between dopamine and attentional control operate in pediatric populations, where genetic variations in D2Rs are associated with ADHD symptoms (Moro et al. 2019) and where optimal dopaminergic intervention may differ from adults.

### Conclusion

In the present study, pharmacologically increasing and decreasing dopamine signalling in the striatum in the same individuals both reduced levels of IOR, supporting a non-linear relationship between striatal dopamine levels and IOR. We propose that these effects emerged from shifts in the balance between attention flexibility and stability, mediated by dopaminergic frontostriatal connections. Such findings have implications for IOR itself, demonstrating the importance of striatal dopamine for attentional inhibition, while also contributing to our understanding of optimal dopaminergic function across multiple cognitive domains. However, there may also be wider implications for the treatment of dopaminergic disorders, particularly the need to consider individual baseline dopamine levels, genetic factors, and circuit-level integrity when designing therapeutic interventions. Future research should investigate the roles of other dopamine receptor subtypes, incorporate polygenic approaches to individual differences, and explore non-pharmacological interventions targeting the same frontostriatal circuits that mediate IOR.

## Supplementary Information

Below is the link to the electronic supplementary material.

Supplementary Material 1

## Data Availability

The datasets generated and analysed during the current study are not publicly available due to ethical and GDPR-related restrictions but are available from the corresponding author on reasonable request.
