# Supplementary material for: The effects of increasing and decreasing dopamine on inhibition of return: a psychopharmacological investigation
Source: Exp Brain Res. 2026 Sep 27;244(11):205. doi: 10.1007/s00221-026-07355-1 (PMC13616838; doi:10.1007/s00221-026-07355-1)
Supplement: Supplementary file 1 [file 221_2026_7355_MOESM1_ESM.docx]

## S1 Supplementary Materials

### S1.1 Individual Reaction Time Distributions

Figure 3 in the main manuscript presents group-level reaction time patterns across drug conditions and trial types. For readers interested in examining individual participant variability underlying these group effects, Supplementary Figure S1 provides a detailed visualisation of the full distribution of reaction times for each condition, with individual data points overlaid on half-violin plots and box plots. Outlier analysis using the 1.5×IQR criterion confirmed that no participant exceeded outlier thresholds in any condition.

**Fig. S1:** Reaction times by drug condition and trial type. Half-violin plots show the distribution of individual participant reaction times, with box plots indicating median, interquartile range, and individual data points overlaid. Changes in striatal dopamine (increase and decrease with D2 agonist and antagonist, respectively) reduce cued reaction times. Neutral trials do not differ between conditions.

### S1.2 Speed-accuracy Trade-off

To assess the possibility of a speed-accuracy trade-off, a repeated measures ANOVA was conducted using percentage incorrect rates for the different IOR conditions (cued and uncued) and dopamine manipulations (agonist, antagonist, and placebo). See Table S1 for descriptive statistics. None of the main effects or the interaction were significant (see Table S2). As such, no speed-accuracy trade-off was apparent.

 **Table S1:** Mean percentage incorrect rates and standard deviations (SDs) for all conditions.

**Table S2:** Results of ANOVA comparing percentage incorrect rates for each inhibition of return (IOR) cue condition and dopamine manipulation.

### S1.3 Proportional IOR

As the active drugs both significantly reduced RTs across task conditions, proportional IOR scores were calculated for each participant and each visit to ensure that the observed differences in IOR magnitudes were not caused by drugs generally speeding responses, thus reducing the relative difference between cued and uncued RTs. Proportional IOR was calculated as IOR magnitude divided by grand mean RT (calculated as the average RT across all responses for each participant and visit), hence providing IOR as a percentage of overall RT. This normalisation approach is analogous to the coefficient of variation sometimes used in RT research to control for baseline differences in response speed (e.g., Epstein et al., 2011).

Proportional IOR scores were analysed using a one-way repeated measures ANOVA with dopamine manipulation as the within-subjects factor (agonist, antagonist, placebo). The main effect of dopamine manipulation was significant, F(2, 54) = 7.05, *p* = .002. Planned pairwise comparisons demonstrated significantly smaller proportional IOR magnitudes in both the agonist (M = 5.1%) and antagonist (M = 4.3%) conditions compared to placebo (M = 6.7%): agonist vs placebo, t(27) = -2.59, *p* = .015; antagonist vs placebo, t(27) = -3.15, *p* = .004. There was no significant difference between agonist and antagonist proportional IOR scores, t(27) = 1.47, *p* = .153. These findings replicate the pattern of results found with non-proportional IOR magnitude, supporting that the drug effects on IOR reflect genuine attentional modulation rather than artifacts of general response slowing.

**Figure S2**: Proportional IOR magnitude by drug condition. Box plots show distribution of individual participant proportional IOR scores. Black points represent mean proportional IOR for each condition, connected by lines. Replicates the pattern observed in Manuscript Figure 4).

**Reference:**

Epstein, J. N., Langberg, J. M., Rosen, P. J., Graham, A., Narad, M. E., Antonini, T. N., Brinkman, W. B., Froehlich, T. E., Simon, J. O., & Altaye, M. (2011). Evidence for higher reaction time variability for children with ADHD on a range of cognitive tasks including reward and event rate manipulations. *Neuropsychology*, 25(4), 427.
